# Trends in treatment and survival of patients with nonresected, nonmetastatic pancreatic cancer: A population‐based study

**DOI:** 10.1002/cam4.1750

**Published:** 2018-09-06

**Authors:** Lydia G. M. van der Geest, Casper H. J. van Eijck, Bas Groot Koerkamp, Valery E. P. P. Lemmens, Olivier R. Busch, Pauline A. J. Vissers, Johanna W. Wilmink, Marc G. Besselink, the Dutch Pancreatic Cancer Group

**Affiliations:** ^1^ Department of Research Netherlands Comprehensive Cancer Organisation (IKNL) Utrecht The Netherlands; ^2^ Department of Surgery Erasmus Medical Center Rotterdam The Netherlands; ^3^ Department of Public Health Erasmus Medical Center Rotterdam The Netherlands; ^4^ Department of Surgery Academic Medical Center University of Amsterdam Amsterdam The Netherlands; ^5^ Department of Medical Oncology Academic Medical Center University of Amsterdam Amsterdam The Netherlands

**Keywords:** chemotherapy, nonresected, pancreatic adenocarcinoma, survival

## Abstract

**Background:**

Nonresected, nonmetastatic (NR‐M0) pancreatic cancer involves both locally advanced pancreatic cancer and patients who did not undergo resection due to poor health status or patient preference. This study investigates nationwide trends of characteristics, treatment, and survival of patients with NR‐M0 pancreatic cancer.

**Methods:**

From the Netherlands Cancer Registry, all patients diagnosed with pancreatic cancer between 2006 and 2014 were selected. Chemotherapy and overall survival (OS) of NR‐M0 patients were evaluated for 3‐year time periods and 2 age groups using chi‐square tests for trend and Cox proportional hazard regression analysis.

**Results:**

Of 18 234 patients, 33% had NR‐M0 pancreatic cancer, which decreased over time (in consecutive 3‐year periods: 38%‐33%‐28%, *P *<* *0.001). Of 5964 NR‐M0 patients, 52% was over 75 years of age, 16% received chemotherapy, and median OS was 5.1 months. Chemotherapy use increased over time in younger patients (<75 years: from 23 to 36%, *P*‐trend < 0.001, ≥75 years: 3% to 4%, *P*‐trend = 0.053). In multivariable survival analysis, elderly age, low SES, nonconfirmed cancer, stage II‐III disease, and earlier years of diagnosis were independently associated with a worse OS. Age of patients who received chemotherapy increased over time (median 62‐66 years) and median OS was 10.4 months without significant differences between time periods (*P* = 0.177) or age groups (*P* = 0.207).

**Conclusions:**

Overall survival of NR‐M0 pancreatic cancer remains poor which is partly related to advanced age of many patients. Despite an increase, chemotherapy is infrequently used. Future research should investigate to what extent the more widespread use of chemotherapy could improve survival in relation to age‐related morbidity.

## 1 INTRODUCTION

Pancreatic cancer remains one of the most lethal cancers with a 5‐year survival rate of 5‐7%.^1^, ^2^ Since symptoms usually emerge late, about 50%‐60% of patients are diagnosed with metastatic disease.^3^, ^4^ Only 10%‐20% of patients have resectable disease. The intermediate group of 30%‐40% generally is referred to as locally advanced pancreatic cancer (LAPC).^5^, ^6^ Nonresected patients in cancer registries have metastatic or unresectable disease diagnosed at imaging or at time of surgical exploration or are ineligible for surgery due to a poor health status or patient preference. As a result of increased resection rates for pancreatic cancer,^7^, ^8^ characteristics of the patient group with nonresected, nonmetastatic (NR‐M0) disease may have changed.

For patients with pancreatic cancer not undergoing resection, chemotherapy is the main treatment modality. In patients with metastatic disease, population‐based studies have shown that the administration of palliative chemotherapy steeply increased in the past decades.^3^, ^9^, ^10^ Notably, this increased use of chemotherapy was found in the gemcitabine era, and thus, before the studies on FOLFIRINOX (5‐fluorouracil, leucovorin, irinotecan, and oxaliplatin) and nab‐paclitaxel plus gemcitabine reported favorable results compared with gemcitabine alone.^11^, ^12^ No randomized controlled trials have yet been published on these chemotherapy schemes in patients with LAPC. Despite a lack of randomized studies, an increased use of chemotherapy may also be found in patients with NR‐M0 disease.

Population‐based data on treatment and survival of patients with LAPC or NR‐M0 disease are scarce.^13^ In addition, little is known about survival of elderly patients with NR‐M0 disease, with or without chemotherapy.

Therefore, the aim of this nationwide study was to investigate time trends in characteristics, treatment, and survival of patients with NR‐M0 pancreatic cancer.

## 2 METHODS

### 2.1 Data collection

The Netherlands Cancer Registry (NCR) records data on all patients with newly diagnosed cancer in the Netherlands, a country with 17 million inhabitants. Since 1989, newly diagnosed malignancies are notified to the NCR by the automated pathological archive (PALGA), supplemented with data from the National Registry of Hospital Discharge Diagnoses. Completeness is estimated to be at least 95%. Trained registrars in all Dutch hospitals routinely extract data on patient, tumor, and treatment characteristics. Tumor location and histology are registered according to the International Classification of Diseases for Oncology (ICD‐O‐3).^14^ The tumour‐node‐metastasis (TNM) staging classification is used (6th edition in 2003‐2009,^15^ 7th edition in 2010‐2016^16^) for pathologically confirmed malignancies, while in other cases, a 1‐digit extend of disease (EoD) is recorded. From 2012 onwards, TNM was recorded for all patients. Actual vital status (dead or alive, date of death or emigration) is obtained by periodically linking the NCR to the Municipal Personal Records Database which keeps record on the vital status of all Dutch inhabitants.

### 2.2 Patients

From the NCR, all patients were selected who were diagnosed with pancreatic (ductal) adenocarcinoma between 2006 and 2014 (ICD‐O‐3 C25, morphology codes 8010, 8012, 8020, 8140,8141, 8260, 8310, 8440, 8480, 8481, 8490, 8500, 8560, or a nonconfirmed supposed adenocarcinoma). Patients diagnosed at autopsy, younger than 18 years or residing abroad, were excluded. The total population was divided into three groups: resected, nonresected nonmetastatic (NR‐M0), and metastatic pancreatic cancers. Since this division was based on findings of imaging and surgical exploration, a number of patients with nonresected disease underwent a laparotomy or laparoscopy (11% of NR‐M0 patients in 2012‐2014). The intermediate group of NR‐M0 patients was the focus of the present study.

The study period was evenly divided into three 3‐year periods: 2006‐2008, 2009‐2011, and 2012‐2014. Patients were divided into two age groups: younger patients <75 years and elderly patients ≥75 years at diagnosis. Comorbidity was recorded regionwide in 2 out of 9 Dutch cancer regions (16% of all patients) according to a slightly modified version of the Charlson classification. Serious comorbid conditions included chronic obstructive pulmonary diseases, cardiovascular diseases, cerebrovascular diseases, digestive tract diseases, diabetes mellitus, and other serious diseases. The number of comorbidities was categorized into three groups (0, 1, and ≥2). In addition, due to the nature of the NCR, information on previous malignancies was available in all patients. Furthermore, socioeconomic status (SES)^17^ was based on reference data from The Netherlands Institute for Social Research. Social deprivation scores were derived from data on income, education, and occupation per 4‐digit postal code and were broken into three SES categories (high: 1st‐3rd, intermediate: 4th‐7th, and low: 8th‐10th deciles). Both types of information on tumor stage (TNM and EoD) were combined into one summary stage: (a) “localized”: tumor confined to the pancreas (TNM I); (b) “nonlocalized”: “tumor extension into adjacent organs or tissues and/or into regional lymph nodes” (TNM II‐III); (c) “metastatic”: distant metastasis (TNM IV); and (d) unknown stage. In the period 2012‐2014, a distinction between stage II (T3/N1M0) and stage III (T4M0) could be made. Registered treatments comprise tumor resection, chemotherapy, and/or local treatment such as radiotherapy applied for stage at diagnosis. No information was available about type of chemotherapy treatment. Survival time was calculated from the date of diagnosis to the date of death or emigration. Patients who were alive on 1 February 2017 were censored (1.6%). To investigate early mortality after diagnosis, 30‐ and 90‐day mortality of any cause after date of diagnosis were calculated.

### 2.3 Statistical analysis

Chi‐square tests for trend were used to analyze characteristics and treatment of the NR‐M0 patients in consecutive 3‐year periods. A two‐sided *P*‐value <0.05 was considered statistically significant. To evaluate overall survival of NR‐M0 patients, Kaplan‐Meier analyses and log‐rank tests were used, as well as univariable and multivariable Cox proportional hazard analyses. In multivariable models, a backward stepwise selection was used with a *P* > 0.10 for removal of variables in likelihood ratio tests. Characteristics that were included (if applicable) were time periods, age, sex, history of cancer, SES, pathological confirmation of cancer, tumor location, summary tumor stage, chemotherapy, and local treatment. Sensitivity analyses were performed using regionwide data to investigate associations of the number and type of comorbid conditions (adjusted for predictors derived from the multivariable model in all patients). STATA/SE (version 14.0; STATA Corp., College Station, TX, USA) was used in all analyses.

## 3 RESULTS

### 3.1 All patients

Median age of 18 234 patients diagnosed with pancreatic cancer in 2006‐2014 was 71 years and 37% was 75 years or older. Pathology confirmation of pancreatic cancer occurred less frequently in patients with nonmetastatic disease (62% vs 69% in metastatic disease, *P* < 0.001). Metastatic disease was present in 9934 (54%) of patients, and 2336 (13%) of patients underwent tumor resection. The remaining 5964 (33%) patients had nonresected, nonmetastatic (NR‐M0) pancreatic cancer. Compared with patients with resected and metastatic cancer, patients with NR‐M0 pancreatic cancer were older (median 75 years vs 67 and 69 years, respectively) and had an intermediate overall survival (median 5.1 months [95% confidence interval: 4.9‐5.2 months] vs 17.5 and 2.3 months, respectively).

**Figure 1:**
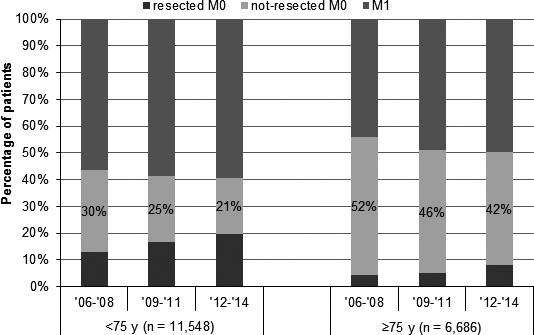
Distribution of resected, nonresected nonmetastatic (NR‐M0), and metastatic (M1) pancreatic cancer, by periods within younger and elderly age categories (both *P* < 0.001)

Both tumor resection and diagnosis of metastatic disease increased over time. As a result, NR‐M0 pancreatic cancer decreased from 2052 (38%) patients in 2006‐2008 to 2026 (33%) in 2009‐2011 and 1886 (28%) in 2012‐2014 (*P*‐trend < 0.001). This time trend was found within younger and elderly age groups alike, as was shown in Figure 1.

### 3.2 Patients with NR‐M0 pancreatic cancer

Overall, 52% of 5964 patients with NR‐M0 pancreatic cancer was aged 75 years and older, with a significant increase over time (50%, 52%, and 54%, *P*‐trend = 0.008; Table 1). Pathological confirmation of cancer occurred in 47% of NR‐M0 patients and increased over time in younger patients only. Arterial involvement was found in 39% of NR‐M0 patients (TNM stage III, 2012‐2014).

**Table 1:** Characteristics of patients with nonresected, nonmetastatic (NR‐M0) pancreatic carcinoma, by time periods within younger and elderly age groups

	All patients					Patients younger than 75 y					Patients 75 y and older				
	All	2006‐2008	2009‐2011	2012‐2014	Chi‐square *P*‐trend	All	2006‐2008	2009‐2011	2012‐2014	Chi‐square *P*‐trend	All	2006‐2008	2009‐2011	2012‐2014	Chi‐square *P*‐trend
N	5964 (%)	2052%	2026%	1886%		2872 (%)	1035%	965%	872%		3092 (%)	1017%	1061%	1014%	
Pathological confirmation															
Confirmed	2790 (47)	45	46	49	0.022	1874 (65)	63	63	71	<0.001	916 (30)	28	30	30	0.208
Not confirmed	3174 (53)	55	54	51		998 (35)	37	37	29		2176 (70)	72	70	70	
Primary tumor															
Head of pancreas	4499 (75)	77	76	74	0.098	2057 (72)	73	73	68	0.014	2442 (79)	80	79	79	0.860
Body or tail	784 (13)	12	12	16		464 (16)	15	15	18		320 (10)	9.1	8.9	13	
Overlapping/NOS	681 (11)	11	12	11		351 (12)	11	12	14		330 (11)	11	12	8.3	
Summary stage															
Localized	1467 (25)	26	26	22	0.595	352 (12)	15	12	9.5	0.260	1115 (36)	37	39	32	0.631
Nonlocalized	3789 (64)	59	64	67		2287 (80)	75	80	84		1502 (49)	43	49	53	
Unknown	708 (12)	15	10	11		233 (8)	9.8	7.8	6.5		475 (15)	20	12	15	
TNM stage I‐II‐X				61					43					77	
TNM stage III				39					57					23	
Chemotherapy (%yes)	967 (16)	13	17	19	<0.001	858 (30)	23	31.5	36	<0.001	109 (3.5)	2.8	3.5	4.3	0.053
Local therapy (%yes)^a^	324 (5.4)	5.5	4.2	6.7	0.121	288 (10)	10	7.8	13	0.074	36 (1.2)	1.0	0.9	1.6	0.212
Deceased within 30 d of diagnosis (%yes)	739 (12)	12	12	13	0.275	191 (6.7)	6.5	6.3	7.2	0.529	548 (18)	17	18	18	0.704
Deceased within 90 d of diagnosis (%yes)	1978 (33)	33.2	32.8	33.51	0.838	697 (24)	25	24	24	0.938	1281 (41)	42	41	41	0.761
Median OS (95% CI) in mo	5.1 (4.9‐5.2)	4.9 (4.6‐5.2)	5.1 (4.8‐5.5)	5.1 (4.8‐5.5)	0.088 b	6.3 (6.0‐6.6)	5.9 (5.5‐6.4)	6.7 (6.0‐7.1)	6.4 (6.1‐6.7)	0.052 b	3.9 (3.7‐4.1)	3.8 (3.5‐4.2)	3.9 (3.6‐4.4)	4.0 (3.6‐4.3)	0.322 b

CI, confidence interval; NOS, not otherwise specified; OS, overall survival.

a For example, conventional radiotherapy, SBRT, RFA, and IRE.

b Log‐rank test.

Only 16% (967/5964) of patients with NR‐M0 disease received chemotherapy, with an increase in consecutive 3‐year periods from 13%, 17%, to 19% (*P*‐trend < 0.001), particularly in younger patients (<75 years: from 23% to 36%, *P*‐trend < 0.001, Table 1). Of patients over 75 years, only 3.5% were treated with chemotherapy (2.8% to 4.3%, *P*‐trend = 0.053). In addition, 5.4% of patients received local therapy such as radiotherapy or (sporadic) ablative treatments (in consecutive periods: 5.5%‐4.2%‐6.7%, respectively, *P*‐trend = 0.121).

At time of 90‐days after diagnosis, 33% (1978/5964) of patients had died, particularly elderly patients (<75 years: 24%, ≥75 years: 41%, *P* < 0.001). No time trends were found in early mortality (Table 1). One‐ and 2‐year overall survival (OS) of patients with NR‐M0 pancreatic cancer were 18% and 5%, respectively (data not shown). In consecutive 3‐year periods, median OS was 4.9, 5.1, and 5.1 months, respectively (*P* = 0.088, Table 1). For patients aged <75 years and ≥75 years, median OS was 6.3 and 3.9 months, respectively (*P* < 0.001). In the younger age group, a very small improvement of OS was found in the study period (from 5.9 to 6.4 months, *P* = 0.052; ≥75 years: 3.8 to 4.0 months, *P* = 0.322). Furthermore, median OS was 10.4 months in patients who received chemotherapy vs 4.2 months in untreated patients (*P* < 0.001). In the multivariable Cox proportional hazard model, elderly age, low SES, nonconfirmed cancer, nonlocalized disease, and diagnosis in earlier years of the study period were independently associated with a worse OS (Table 2). In a second model including treatment, the increased use of chemotherapy could not completely remove differences between time periods. Among cases with available comorbidity data, only the presence of pulmonary disease was additionally associated with a worse OS (n = 864, HR = 1.29, 95% CI 1.06‐1.59).

**Table 2:** Univariable and multivariable Cox proportional hazards analyses predicting overall survival of patients with nonresected, nonmetastatic (NR‐M0) pancreatic cancer

	N	MS (mo)	Univariable analysis		Multivariable analysis		Multivariable analysis including treatment	
			HR (95% CI)	*P*‐value	HR (95%CI)	*P*‐value	HR (95%CI)	*P*‐value
Overall	5964	5.1						
Period of diagnosis								
2005‐2008	2052	4.9	Ref	0.090	Ref		Ref	
2009‐2011	2026	5.1	0.93 (088‐0.99)		0.93 (0.87‐0.99)	0.020	0.94 (0.88‐1.00)	0.043
2012‐2014	1886	5.1	0.97 (0.89‐1.02)		0.94 (0.88‐1.00)	0.067	0.99 (0.93‐1.05)	0.664
Age (y)								
<75	2870	6.3	Ref	<0.001	Ref		Ref	
≥75	3092	3.9	1.36 (1.29‐1.43)		1.39 (1.31‐1.47)	<0.001	1.21 (1.14‐1.28)	<0.001
Sex								
Male	2745	5.1	Ref	0.249				
Female	3219	5.1	1.03 (0.98‐1.08)					
History of cancer								
No	4956	5.1	Ref	0.008				
Yes	1008	4.5	1.10 (1.03‐1.18)					
Socioeconomic status								
High	1718	5.4	Ref	0.001	Ref		Ref	
Intermediate	2405	4.9	1.03 (0.96‐1.09)		1.03 (0.96‐1.09)	0.416	1.01 (0.95‐1.08)	0.762
Low	1841	4.7	1.12 (1.05‐1.20)		1.12 (1.05‐1.20)	0.001	1.11 (1.04‐1.19)	0.002
Pathological confirmation								
Confirmed	2790	6.0	Ref	<0.001	Ref		Ref	
Not confirmed	3174	4.1	1.18 (1.12‐1.24)		1.09 (1.03‐1.16)	0.004	0.99 (0.94‐1.05)	0.827
Primary tumor location								
Head of pancreas	4499	5.1	Ref	0.108				
Body or tail	784	5.2	0.93 (0.86‐1.00)					
Overlapping/NOS	681	4.7	1.02 (0.94‐1.10)					
Summary stage								
Localized	1467	4.9	Ref	<0.001	Ref		Ref	
Nonlocalized	3789	5.5	1.04 (0.98‐1.11)		1.27 (1.18‐1.36)	<0.001	1.34 (1.25‐1.44)	<0.001
Unknown	708	3.5	1.25 (1.14.1.37)		1.31 (1.19‐1.43)	<0.001	1.27 (1.16‐1.39)	<0.001
Chemotherapy								
No	4997	4.2	Ref	<0.001	X		Ref	
Yes	967	10.4	0.53 (0.49‐0.56)				0.56 (0.52‐0.61)	<0.001
Local therapy								
No	5640	4.7	Ref	<0.001	X		Ref	
Yes	324	11.3	0.55 (0.49‐0.62)				0.77 (0.68‐0.87)	<0.001

MS, median survival.

### 3.3 Patients with NR‐M0 disease who received chemotherapy

Median age of 967 patients with NR‐M0 pancreatic cancer who received chemotherapy was 64 years (range 34‐85 years) and increased in consecutive 3‐year periods (median age 62, 63, 66 years, respectively, *P* = 0.007). Of these treated patients, as many as 17% did not undergo pathological confirmation of cancer (Table 3), which decreased over time (19%, 20%, 12% of treated patients in consecutive time periods, *P* = 0.015). Most patients receiving chemotherapy had locally advanced disease (stage II‐III: 87%, 91%, and 94% in consecutive 3‐year periods, *P* = 0.013; stage III: 67% of 357 treated patients diagnosed in 2012‐2014). One‐ and 2‐year survival were 41% and 11%, respectively. Median OS of treated patients was 10.5, 9.6, and 10.8 months in consecutive time periods (*P* = 0.177; Table 3) and did not differ significantly between age groups (<75 years: 10.6 and ≥75 years: 9.2 months, *P* = 0.207; data not shown).

**Table 3:** Characteristics of patients with nonresected, nonmetastatic (NR‐M0) pancreatic carcinoma receiving chemo(radio)therapy, by time periods

	All patients	2006‐2008	2009‐2011	2012‐2014	Chi‐square
	N = 967%	N = 269%	N = 341%	N = 357%	*P*‐trend
Median age (range)	64 (34‐85)	62 (34‐83)	64 (36‐84)	66 (38‐85)	0.007
Pathological confirmation					
Confirmed	807 (83)	81	80	88	0.015
Not confirmed	160 (17)	19	20	12	
Primary tumor					
Head of pancreas	645 (67)	68	68	64	0.102
Body or tail	206 (21)	22	17	24	
Overlapping/NOS	116 (12)	9.7	14	11	
Summary stage					
Localized	62 (6.4)	9.7	5.3	5.0	0.013
Nonlocalized	878 (91)	87	91	94	
Unknown	27 (2.8)	3.7	3.8	1.1	
TNM stage I‐II‐X	117 (33)			33	
TNM stage III	240 (67)			67	
Local therapy (%yes)^a^	247 (26)	36	19	24	<0.001
Deceased within 90 days of start chemotherapy (%yes)	101 (14)	11	16	13	0.342
Median OS (95%CI) in mo	10.4 (9.9‐10.9)	10.5 (9.4‐11.8)	9.6 (8.7‐10.7)	10.8 (10.2‐11.5)	0.177^b^

CI, confidence interval; NOS, not otherwise specified; OS, overall survival.

a For example, conventional radiotherapy, SBRT, RFA, and IRE.

b Log‐rank test.

## 4 DISCUSSION

One‐third of patients with pancreatic cancer in the Netherlands (2006‐2014) had nonresected, nonmetastatic (NR‐M0) disease. At least half of these nearly 6.000 NR‐M0 patients were over 75 years of age and two‐fifth of patients had stage III disease. The median overall survival of NR‐M0 pancreatic cancer was 5.1 months. Only 16% of NR‐M0 patients received chemotherapy with a median survival of 10.4 months. In the course of our study, a 50% increase in chemotherapy use was found within the younger age group (<75 years), though without significant improvement of survival.

In the past decade, the resection rate for pancreatic cancer in the Netherlands has increased,^7^, ^8^ whereas detection of metastatic disease also increased (stage migration).^3^ Consequently, the proportion of patients in the remaining group with NR‐M0 pancreatic cancer decreased until less than one‐third in 2012‐2014, while age of patients with NR‐M0 disease increased. In addition, only 40% of the NR‐M0 patient group in 2014‐2016 had stage III disease. Particularly in the remaining 60% of NR‐M0 pancreatic cancer patient stage I‐II, elderly patients were overrepresented (≥75 years: 68%). Several retrospective studies suggested underutilization of surgical treatment in elderly patients with localized pancreatic cancer.^18^, ^19^, ^20^ However, many NR‐M0 patients die soon after diagnosis; in our study, 41% of patients over 75 years died within 90 days. Though in a previous study comorbidity of (elderly) patients was not associated with the application of pancreatic surgery,^18^ a poor general health status at time of diagnosis may have precluded surgical treatment. Accurate identification of a poor health status of patients is of utmost importance for optimal treatment decision making.^21^

Most patients not eligible for pancreatic surgery due to a poor performance status are also not candidates for chemotherapy. In the current study period in the Netherlands, the administration of chemotherapy to NR‐M0 patients was very limited (16%), which can largely be attributed to the high number of elderly patients with early‐stage disease (77% of stage I‐II and 43% of stage III patients were aged ≥75 years). In addition, a restraint of medical oncologists to give chemotherapy to elderly patients and patient preferences could have added to limited chemotherapy use. Also in the subgroup of patients with stage III disease, chemotherapy use in our study (34% in 2012‐2014) was limited compared with population‐based studies in the United States (>50%).^13^, ^22^ Similar data on chemotherapy use were found in a previous study of our group in patients with metastatic pancreatic cancer.^10^

Despite a major increase in chemotherapy use in NR‐M0 patients under 75 years in the current study, overall survival hardly improved. However, the study period mainly covers the gemcitabine era, chemotherapy use and response rates may simply be too low to show a survival improvement in all NR‐M0 pancreatic cancer patients. Possibly, increasing prescription of more effective chemotherapy schemes such as FOLFIRINOX and nab‐paclitaxel with gemcitabine may affect overall survival in years following the current study period. In addition, the age of chemotherapy‐treated patients in our study has substantially risen from median 62 to 66 years, though still few elderly patients received chemotherapy (≥75 years: 11%). A careful selection and better support of elderly patients for chemotherapy treatment is therefore relevant and can be facilitated by the use of geriatric assessment tools.^23^

Strikingly, pathological confirmation of cancer in our study was lacking in one in six NR‐M0 patients who received chemotherapy. Because a misdiagnosis cannot be ruled out,^24^ pathological confirmation before chemotherapy is highly recommended.^25^ The absence of pathological confirmation in treated patients is worrisome and requires further attention in multidisciplinary team discussions in the Netherlands.

In recent systematic reviews of nonrandomized studies with LAPC patients only, approximately one quarter of patients could undergo resection after neoadjuvant chemo(radio)therapy^26^, ^27^ and overall survival of patients receiving FOLFIRINOX^28^ with or without resection was 24 months,^26^ which was comparable with survival of patients with initially resectable pancreatic cancer. In the current nationwide study, chemotherapy was combined with radiotherapy in only a minority of chemotherapy‐treated NR‐M0 patients.^25^, ^29^ Re‐evaluation of NR‐M0 patients after several months of chemo(radio)therapy may be worthwhile to identify patients for possible resection or eligibility for other treatments directed at local tumor control. An experienced multidisciplinary team or expert panel can provide in this need.

This study has several limitations that are related to the retrospective data that were used. Firstly, due to the available notification sources, the NCR is at risk of incompleteness of pancreatic cancer in elderly patients.^30^ Therefore, chemotherapy use and survival of elderly NR‐M0 patients may be slightly overestimated in our study, while early mortality may be underestimated. Survival may also be slightly overestimated because some patients of the large group without histological confirmation of pancreatic cancer were incorrectly diagnosed.^24^ Despite these limitations, the available unselected data of an often neglected group of pancreatic cancer patients revealed important findings about trends in everyday clinical practice. Secondly, survival trends of NR‐M0 patients in the course of the study period must be interpreted with caution as a result of changing characteristics of this subgroup and possible residual confounding of unmeasured characteristics. Thirdly, although the proportion of patients with stage III disease in our study (12% of all stage I‐IV in 2012‐2014) was comparable with other population‐based studies (7%‐13%),^13^, ^19^, ^31^ staging may be suboptimal in patients who were staged based on imaging only. Locally advanced and metastatic disease was found in a substantial proportion of patients who preoperatively were thought to have resectable disease.^32^, ^33^ Furthermore, TNM staging information cannot discriminate between the currently used categories of resectable, borderline resectable, and irresectable pancreatic cancer, based on the extent of arterial and venous involvement.^34^ Finally, data on comorbid conditions were available in only a subgroup of patients, and no information was available about performance status and quality of life of patients. In the future, the Dutch nationwide PAncreatic CAncer Project (PACAP) will provide more detailed information.^35^

In conclusion, our study showed that the group of NR‐M0 pancreatic cancer patients is heterogeneous, consisting of patients with irresectable tumors due to arterial involvement (stage III) and many patients with advanced age and (supposed) stage I‐II tumors. Despite an increase in the use of chemotherapy in younger patients, overall survival of all patients hardly improved over the described time period.
